# Prospective Association between Multimorbidity and Falls and Its Mediators: Findings from the Irish Longitudinal Study on Ageing

**DOI:** 10.3390/jcm11154470

**Published:** 2022-07-31

**Authors:** Louis Jacob, Jae Il Shin, Karel Kostev, Josep Maria Haro, Guillermo F. López-Sánchez, Lee Smith, Ai Koyanagi

**Affiliations:** 1 Research and Development Unit, Parc Sanitari Sant Joan de Déu, Dr. Antoni Pujadas, 42, Sant Boi de Llobregat, 08830 Barcelona, Spain; josepmaria.haro@sjd.es (J.M.H.); ai.koyanagi@sjd.es (A.K.); 2 Centro de Investigación Biomédica en Red de Salud Mental (CIBERSAM), ISCIII, 28029 Madrid, Spain; 3 Faculty of Medicine, University of Versailles Saint-Quentin-en-Yvelines, 78180 Montigny-le-Bretonneux, France; 4 Department of Pediatrics, Yonsei University College of Medicine, Seoul 03722, Korea; shinji@yuhs.ac; 5 Philipps University of Marburg, 35037 Marburg, Germany; Karel.Kostev@iqvia.com; 6 Division of Preventive Medicine and Public Health, Department of Public Health Sciences, School of Medicine, University of Murcia, 30100 Murcia, Spain; gfls@um.es; 7 Centre for Health, Performance, and Wellbeing, Anglia Ruskin University, Cambridge CB1 1PT, UK; lee.smith@aru.ac.uk; 8 Institució Catalana de Recerca i Estudis Avançats (ICREA), Pg. Lluis Companys 23, 08010 Barcelona, Spain

**Keywords:** multimorbidity, falls, older adults, prospective study, Ireland

## Abstract

This study including older adults from Ireland aimed to analyze the prospective association between multimorbidity and falls and to identify the mediators in this relationship. The present study used data from two consecutive waves of the Irish Longitudinal Study on Ageing (TILDA) survey. Multimorbidity was assessed at Wave 1 (2009–2011) and was defined as the presence of at least two chronic conditions. Falls occurring at Wave 2 (2012–2013) were self-reported. Mediating variables considered were polypharmacy, cognitive impairment, sleep problems, pain, low handgrip strength, difficulty in activities of daily living (ADL), obesity, and underweight. Multivariable binary logistic regression and mediation analysis using the Karlson Holm Breen method were conducted. This study included 6900 adults aged ≥50 years (51.6% women; mean [SD] age 63.1 [8.9] years). Compared to no chronic conditions at baseline, there was a positive and significant association between multimorbidity and falls at follow-up, with ORs ranging from 1.32 (95% CI = 1.06–1.64) for 2 conditions to 1.92 (95% CI = 1.54–2.38) for ≥4 conditions. Pain (23.5%), polypharmacy (13.3%), and difficulty in ADL (10.7%) explained the largest proportion of the multimorbidity-fall relationship. Multimorbidity increased risk for incident falls in older adults from Ireland. Interventions should be implemented to reduce fall risk in people with multimorbidity, especially targeting the identified mediators.

## 1. Introduction

Based on the definition of the World Health Organization (WHO), falls correspond to events resulting in an individual coming to rest on the ground or the floor by inadvertence [1]. Falls are frequent in the general population and highest among older adults, with the 2017 global age-standardized prevalence being approximately 5200 cases per 100,000 persons [2]. In addition, falls are associated with impaired mental wellbeing [3], low quality of life [4], and increased mortality [5], while the direct and indirect costs related to the management of falls are substantial [6,7]. Thus, the identification of risk factors for falls, especially among the older population, is of prime importance to inform targeted intervention and policy.

One understudied but potentially important risk factor for falls is multimorbidity. Multimorbidity is defined as the coexistence of at least two conditions [8], and is highly prevalent among the elderly [9]. Multimorbidity may increase the risk of falls via factors such as pain [10,11], polypharmacy [12,13], disability [14,15,16], and sleep problems [17,18]. In the past few years, several studies have investigated the potential association between multimorbidity and falls, and these studies reported a positive multimorbidity–fall relationship [19,20,21,22,23,24]. For example, a population-based prospective cohort study of 10,594 middle-aged women from Finland showed that the number of chronic disorders was positively associated with the risk of falls [21]. It was further observed in another cross-sectional study, including 16,357 older adults living in Canada, that the risk of falls was significantly higher in those with multiple conditions than in their counterparts without any conditions [20]. Although the findings of the previous studies are of particular importance, most of these studies were of cross-sectional nature, and thus, little is known on the directionality of the association between multimorbidity and falls. Moreover, the only two longitudinal studies focusing on the multimorbidity–fall relationship were conducted in specific groups of individuals (i.e., postmenopausal women [21] and older adults with back pain [24]), and their results may not be generalizable to the entire older population. Finally, no research has yet studied the potential mediating role of several factors such as pain, polypharmacy, and cognitive impairment in this association. In this context, more research on the multimorbidity–fall relationship is warranted in order to better design preventive measures for falls among people with multimorbidity.

Therefore, the goals of this prospective study, including adults aged ≥50 years from Ireland, were to investigate the association between multimorbidity and incident falls, and to identify mediators in this relationship.

## 2. Materials and Methods

### 2.1. The Survey 

Data from two consecutive waves of the Irish Longitudinal Study on Ageing (TILDA) survey were used for this study. The methodology of the survey has been extensively described in the literature [25,26]. Briefly, the survey was undertaken by Trinity College Dublin, and included adults aged ≥50 years from private households in Ireland. Nationally representative samples were obtained using clustered random sampling. Wave 1 (i.e., baseline survey) was conducted in October 2009–February 2011, and Wave 2 in April 2012–January 2013. People living in institutions, those affected by dementia, and those with severe cognitive impairment limiting their ability to provide written informed consent were excluded from Wave 1. The majority of data were obtained through interviews conducted by trained staff using a computer. Sensitive data (e.g., alcohol consumption) were obtained using a self-completed questionnaire returned following the interview. The response rate was 62% and 86% for Wave 1 and Wave 2, respectively. Sampling weights were generated based on the statistics of the Quarterly National Household Survey 2010, and the estimation of these weights relied on sex, age, and educational attainment. Finally, ethical approval for the survey was obtained from the Ethics Committee of the Faculty of Health Sciences of Trinity College Dublin, while each participant provided written informed consent.

### 2.2. Falls at Wave 2 (Dependent Variable)

Falls at Wave 2 were assessed with the yes/no question “Have you fallen since the last interview?”

### 2.3. Chronic Conditions and Multimorbidity (Independent Variables)

We considered all chronic conditions for which data were available in the survey. The question “Has a doctor ever told you that you have any of the conditions on this card?” was used to assess the presence of chronic physical conditions at Wave 1. There were 14 conditions listed on the card: arthritis, asthma, cancer, chronic lung disease (i.e., chronic bronchitis or emphysema), cirrhosis, diabetes, eye disease (i.e., age-related macular degeneration, cataracts, glaucoma, or other eye disease), heart disease (i.e., abnormal heart rhythm, angina, congestive heart failure, heart attack, heart murmur, or other heart disease), high cholesterol, hypertension, osteoporosis, stomach ulcer, stroke, and varicose ulcer. In addition, we also included anxiety and depression in the list of chronic conditions. The assessment of anxiety symptoms relied on the anxiety subscale of the Hospital Anxiety and Depression Scale (HADS-A), with a score of ≥8 corresponding to a positive screen for generalized anxiety disorder [27]. Previous research has shown that the sensitivity and the specificity of this cut-off for the screening of generalized anxiety disorder is 89% and 75%, respectively [28]. Depressive symptoms were evaluated with the 20-item Center for Epidemiologic Studies Depression (CES-D), a scale focusing on past-week symptoms, and a cut-off score of ≥16 corresponded to a positive screen for depression [29]. The sensitivity and the specificity of this cut-off for major depression in a community-based sample of older adults were found to be around 100% and 88%, respectively [30]. In line with the commonest definition of multimorbidity [8], multimorbidity corresponded to the presence of at least two conditions. The number of chronic conditions was also used as an ordinal variable with five categories (i.e., 0, 1, 2, 3, and ≥4 conditions).

### 2.4. Mediating Variables

Past literature was used to select variables (i.e., polypharmacy [12,13], cognitive impairment [31,32], sleep problems [17,18], pain [10,11], low handgrip strength [33,34], difficulty in activities of daily living (ADL) [14,15,16], obesity [35,36], and underweight [37,38]), which may act as mediators in the association between multimorbidity and falls. Specifically, these were factors that can be the consequence of multimorbidity and can potentially be the cause of falls. All mediating variables were assessed at Wave 1. Polypharmacy corresponded to the use of ≥5 medications [39]. Cognitive impairment was assessed by the Mini-Mental State Examination (MMSE) [40]. We used a cut-point of <23 to define cognitive impairment, as this cut-off has been validated as being optimal for screening for dementia in Irish community-based samples [41]. Sleep problems were based on a composite sleep score ranging from 0 to 7, with higher scores indicating more sleep problems [42]. This score relied on three questions on the likelihood of dozing off or falling asleep during the day, the frequency of trouble falling asleep, and the frequency of trouble with waking up too early and not being able to fall asleep again. We dichotomized this variable based on the median score of the composite sleep score. Individuals with pain were those answering positively to the question “Are you often troubled with pain?”. Handgrip strength corresponded to the average value of two handgrip measurements of the dominant hand, and low handgrip strength was defined as <30 kg for men and <20 kg for women [43]. Next, participants were asked to indicate if they had difficulties with six types of ADL (i.e., bathing, dressing, eating, getting in or out of bed, using the toilet, and walking). Difficulty in ADL referred to having difficulty with at least one ADL. Finally, obesity and underweight were defined as body mass index ≥30 kg/m^2^ and <18.5 kg/m^2^, respectively, based on measured weight and height.

### 2.5. Control Variables

The selection of control variables relied on past literature [31,44]. All control variables were assessed at Wave 1. Control variables included sex, age (i.e., 50–59, 60–69, 70–79, and ≥80 years), education, marital status (i.e., married/cohabiting, never married, or separated/divorced/widowed), alcohol consumption (i.e., non-drinking, light/moderate drinking, or heavy drinking), and previous falls. Education was classified as: primary (i.e., some primary/not complete, primary, or equivalent); secondary (i.e., intermediate/junior/group certificate or equivalent, leaving certificate, or equivalent); and tertiary (i.e., diploma/certificate, primary degree, or postgraduate/higher degree). Previous falls referred to falls that had occurred in the past 12 months.

### 2.6. Statistical Analysis

The analysis was performed with Stata version 14.2 (Stata Corp LP, College Station, TX, USA). There were 8163 adults aged ≥50 years participating at Wave 1. Data on falls at Wave 2 were available for 6900 individuals, and these participants constituted the final study sample. Differences in sample characteristics between those with and without multimorbidity (i.e., ≥2 chronic conditions) at baseline were tested with Chi-squared tests. The association between the number of chronic conditions or individual chronic conditions at baseline (independent variables) and falls at follow-up (dependent variable) was estimated by multivariable binary logistic regression.

Next, using the *khb* (Karlson Holm Breen) command in Stata [45], a mediation analysis was conducted to quantity the degree to which polypharmacy, cognitive impairment, sleep problems, pain, low handgrip strength, difficulty in ADL, obesity, and underweight at baseline mediate the association between multimorbidity at baseline and falls at follow-up. Based on this method, which can be applied to logistic regression, the total effect of a variable (i.e., the effect unadjusted for the mediator) can be decomposed into direct (i.e., the effect of multimorbidity on falls adjusted for the mediator) and indirect effects (i.e., the mediational effect). The mediating variables were included individually in the models, with the exception of the analysis where all mediating variables were simultaneously included.

All regression analyses, including the mediation analysis, were adjusted for sex, age, education, marital status, alcohol consumption, and previous falls. The regression analysis on individual chronic conditions mutually adjusted for all 16 chronic conditions. The sample weighting and the complex study design with clustering within households were taken into account to obtain nationally representative estimates using the Stata *svy* command. Results are displayed as odds ratios (ORs) and 95% confidence intervals (95% CIs). A *p*-value < 0.050 was considered statistically significant.

## 3. Results

The analytical sample consisted of 6900 individuals aged ≥50 years who participated in Wave 1 and who provided information on falls at Wave 2 (51.6% women; mean [standard deviation] age 63.1 [8.9] years). The sample characteristics are provided in Table 1.

**Figure 1:**
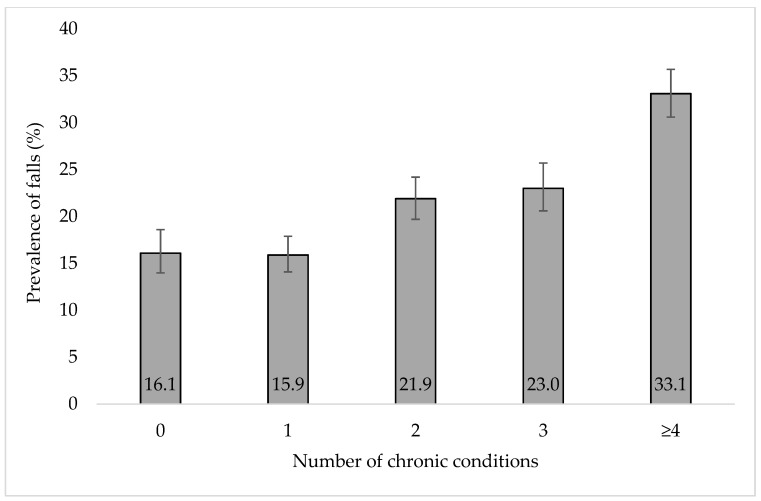
Prevalence of falls at follow-up by number of chronic conditions at baseline. Falls were those which were assessed at Wave 2 and referred to those that occurred since Wave 1. Bars denote 95% confidence intervals. *p*-value < 0.001 based on Chi-squared test.

The prevalence of female sex, older age, lower education, separated/divorced/widowed, no alcohol consumption, previous falls, polypharmacy, sleep problems, pain, low handgrip strength, difficulty in ADL, and obesity were higher among those with multimorbidity (i.e., with ≥2 chronic conditions) than in those without multimorbidity. At baseline, 24.5%, 22.4%, 16.9%, and 20.0% of participants had 1, 2, 3, and ≥4 chronic conditions, respectively, while 21.9% reported at least one fall at follow-up. The prevalence of falls at follow-up increased with the number of chronic conditions at baseline (Figure 1). For example, this prevalence was 16.1% among those without any chronic conditions and 33.1% among those with ≥4 chronic conditions. The prevalence of falls at follow-up among those with and without multimorbidity at baseline was 26.0% and 16.0%, respectively.

The multivariable logistic regression analysis further showed that, compared to no chronic condition at baseline, 2, 3, and ≥4 chronic conditions were associated with 1.32 (95% CI = 1.06–1.64), 1.32 (95% CI = 1.05–1.66), and 1.92 (95% CI = 1.54–2.38) times higher odds for falls at follow-up (Table 2).

**Figure 2:**
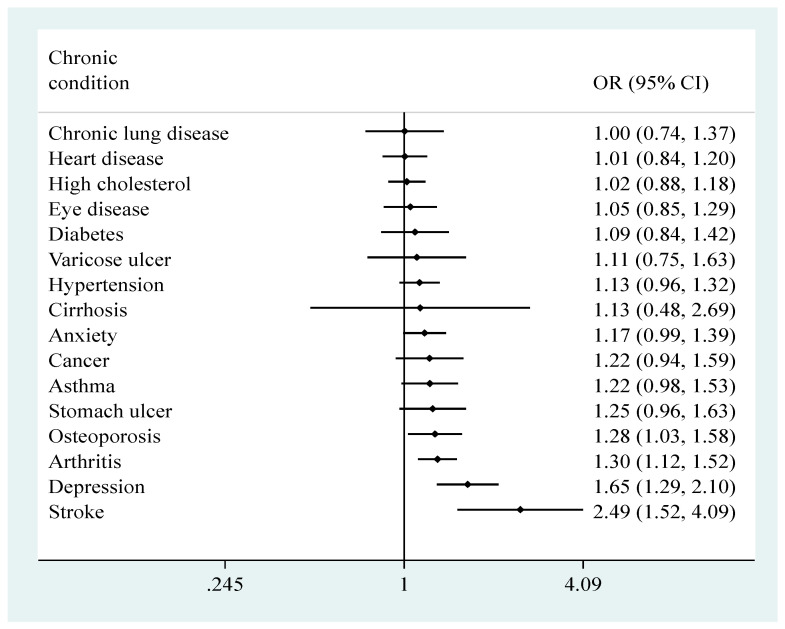
Prospective association between individual chronic conditions at baseline and falls at follow-up estimated by multivariable logistic regression. Abbreviations: OR, odds ratio; CI, confidence interval. Falls were those which were assessed at Wave 2 and referred to those that occurred since Wave 1. The logistic regression model was mutually adjusted for all individual chronic conditions, as well as sex, age, education, marital status, alcohol consumption, and previous falls.

In terms of individual chronic conditions, the adjusted analysis showed that osteoporosis, arthritis, depression, and stroke at baseline were associated with significantly higher risk for falls prospectively, with the OR ranging from 1.28 (95% CI = 1.03–1.58) for osteoporosis to 2.49 (95% CI = 1.52–4.09) for stroke (Figure 2).

Finally, the mediation analysis showed that pain explained the largest proportion of the association between multimorbidity and falls (23.5%), followed by polypharmacy (13.3%), difficulty in ADL (10.7%), sleep problems (9.3%), and obesity (5.1%) (Table 3). All mediators collectively explained 44.5% of the multimorbidity–fall relationship.

## 4. Discussion

### 4.1. Main Findings

This prospective study of 6900 older adults from Ireland found that four individual conditions (i.e., osteoporosis, arthritis, depression, and stroke) were significantly and prospectively associated with falls. Moreover, there was a dose-dependent relationship between the number of chronic conditions and falls, and the OR reached 1.92 for ≥4 disorders (versus no disorders). Finally, pain, polypharmacy, difficulty in ADL, sleep problems, and obesity explained 23.5%, 13.3% 10.7%, 9.3%, and 5.1% of the multimorbidity–fall association, respectively. To the best of the authors’ knowledge, this is the first prospective study analyzing the relationship between multimorbidity and incident falls in the general older population, while it is also the first to quantify the degree to which several mediators may explain this association.

### 4.2. Interpretation of Findings

The association between individual chronic conditions and incident falls may be explained by several different mechanisms. For example, osteoporosis may potentially lead to falls via thoracic kyphosis, quadriceps weakness, and fear of falling [46]. Arthritis may increase risk of falls via pain and muscle weakness [47]. The association between depression and falls can involve fear of falling, decreased fall efficacy, and side effects of antidepressant medications (e.g., impaired gait and decreased balance) [48]. Finally, the association between stroke and incident falls may be explained by reduced muscular tone, hypoesthesia, and hemianopsia [49].

Our finding that multimorbidity leads to an increased risk of falls may partly be explained by the cumulative effects of individual chronic conditions via the mechanisms mentioned above, or through the effects of the identified mediators in our study. Specifically, pain, polypharmacy, difficulty in ADL, and sleep problems were identified as the most important mediators, while obesity was also a significant mediator but to a lesser extent. Chronic pain is frequent in people with multimorbidity [11], while a systematic review and meta-analysis of 21 studies revealed that pain is associated with a significant increase in the risk of falling, and the effects of pain on falls may be mediated by impaired executive function and fear of falling [10]. Polypharmacy is common in patients with multimorbidity as they may receive multiple medications for each individual condition [13]. Polypharmacy in turn increases the risk of falls [12], and this may be due to factors such as orthostatic hypotension and confusion [50,51]. Next, difficulty in ADL may be related to the symptoms or sequalae of chronic diseases (e.g., stroke) and multimorbidity, and physical function limitations are known to increase the risk of falls [15]. In terms of sleep problems, previous research has shown that multimorbidity may lead to a particularly high risk of altered sleep, as sleep problems are common in a wide range of chronic conditions (e.g., sleep-disordered breathing in chronic lung disease and nocturia in diabetes) [17]. In turn, sleep problems may increase risk for falls due to impaired daytime functioning, decreased psychomotor performance, and diminished postural control [18,52]. Finally, a bidirectional association likely exists between multimorbidity and obesity, but multimorbidity may lead to obesity via functional impairment or sedentary behavior [36], while obesity, in turn, may lead to falls through impaired lower-limb muscle quality, increased foot load, and decreased postural control [35].

### 4.3. Public Health Implications and Directions for Future Research

Based on the findings of this prospective study, interventions aiming at the prevention of falls in older people with multimorbidity are urgently warranted. The results of our study suggest that factors such as pain, polypharmacy, difficulty in ADL, and sleep problems should be assessed and addressed in individuals with multimorbidity. Given that side effects of drugs are frequent in older adults, while polypharmacy may increase risk for falls, nonpharmacological interventions should be prioritized for the management of pain (e.g., acupuncture and guided imagery) [53] and sleep problems (e.g., mindfulness and cognitive behavioral therapy) [54]. Regarding polypharmacy, a multidisciplinary approach should be preferred, and regular drug regimen reviews may reduce the risk of inappropriate medication use [55]. Another potentially effective intervention is promotion of physical activity. Indeed, weekly programs combining balance and resistance exercises may help reduce the occurrence of falls as well as improve the management of underlying comorbidities and difficulty in ADL. In terms of future research, more data of longitudinal nature, including intervention studies, are needed for the design of effective strategies to reduce falls among older people with multimorbidity. Furthermore, future studies should assess which combinations of pathologies are associated most with the risk of falling.

### 4.4. Strengths and Limitations

The strengths of this study include the large sample size and the use of prospective nationally representative data. Nonetheless, the study results should be interpreted in the light of several limitations. First, while the present list of chronic conditions included a variety of conditions, there was a lack of information on diseases such as orthostatic hypotension or neuropathy, which can lead to particularly high risk for falls. Thus, the estimates of this study could have differed with a different set of diseases assessed. Second, most of the data used in our study, including data on chronic conditions and falls, were based on self-report. Thus, it is possible that some level of bias (e.g., recall bias) was introduced in our findings. Third, baseline data on multimorbidity and other control or mediating variables were used for the analysis. Consequently, it is possible that some conditions or characteristics of the respondents changed between the two waves. Fourth, Wave 1 was conducted in October 2009–February 2011, while Wave 2 was conducted in April 2012–January 2013. It is possible for the different seasons and survey durations between the two waves to have resulted in different qualities of the survey. Finally, people who were not followed at Wave 2 were more likely to be older, not married/cohabiting, and have lower levels of education at baseline. Thus, it is possible for some level of attrition bias to exist.

## 5. Conclusions

This prospective study of 6900 community-dwelling individuals aged ≥50 years from Ireland revealed that multimorbidity was positively and significantly associated with incident falls. Furthermore, pain, polypharmacy, difficulty in ADL, and sleep problems explained 9.3% to 23.5% of the multimorbidity–fall relationship. Future interventional studies are needed to assess whether addressing the mediators identified in this study can lead to lower risk of falls among those with multimorbidity.

## Figures and Tables

**Table 1:** Sample characteristics (overall and by multimorbidity status).

			Multimorbidity ^1^		
Characteristic	Categories	Total	No (40.7%)	Yes (59.3%)	*p*-Value ^2^
Sex	Female	51.6	43.9	56.9	<0.001
	Male	48.4	56.1	43.1	
Age (years)	50–59	42.1	54.1	33.9	<0.001
	60–69	31.8	29.5	33.4	
	70–79	19.1	12.0	23.9	
	≥80	7.1	4.4	8.8	
Education	Primary	35.4	29.5	39.4	<0.001
	Secondary	44.9	48.6	42.3	
	Tertiary	19.8	21.9	18.3	
Marital status	Married/cohabiting	69.4	74.4	66.0	<0.001
	Never married	9.5	9.9	9.2	
	Separated/divorced/widowed	21.1	15.7	24.8	
Alcohol consumption	Non-drinking	29.2	25.0	31.9	<0.001
	Light/moderate drinking	43.4	43.9	43.0	
	Heavy drinking	27.5	31.1	25.1	
Previous falls	No	80.9	84.5	78.5	<0.001
	Yes	19.1	15.5	21.5	
Polypharmacy	No	79.2	96.2	67.6	<0.001
	Yes	20.8	3.8	32.4	
Cognitive impairment	No	97.3	97.8	97.0	0.123
	Yes	2.7	2.2	3.0	
Sleep problems	Low	60.3	70.3	53.4	<0.001
	High	39.7	29.7	46.6	
Pain	No	63.7	77.4	54.2	<0.001
	Yes	36.3	22.6	45.8	
Low handgrip strength	No	59.0	67.4	53.5	<0.001
	Yes	41.0	32.6	46.5	
Difficulty in ADL	No	91.6	97.2	87.7	<0.001
	Yes	8.4	2.8	12.3	
Obesity	No	65.4	71.7	61.1	<0.001
	Yes	34.6	28.3	38.9	
Underweight	No	99.5	99.3	99.6	0.241
	Yes	0.5	0.7	0.4	

Abbreviation: ADL, activities of daily living. Data are %. All data were obtained at Wave 1. ^1^ Multimorbidity referred to ≥2 chronic conditions. ^2^ *p*-value was estimated using Chi-squared tests.

**Table 2:** Prospective association between the number of chronic conditions at baseline and falls at follow-up estimated by multivariable logistic regression.

Characteristic		OR	95%CI	*p*-Value
Number of chronic	0	1.00		
conditions	1	0.95	[0.76,1.18]	0.630
	2	1.32	[1.06,1.64]	0.015
	3	1.32	[1.05,1.66]	0.018
	≥4	1.92	[1.54,2.38]	<0.001

Abbreviations: OR, odds ratio; CI, confidence interval. The logistic regression model was adjusted for sex, age, education, marital status, alcohol consumption, and previous falls. Falls were those that were assessed at Wave 2 and referred to those that occurred since Wave 1.

**Table 3:** Mediating variables in the prospective association between multimorbidity (i.e., ≥2 chronic conditions) at baseline and falls at follow-up.

	Total Effect		Direct Effect		Indirect Effect		
Mediating Variable	OR [95% CI]	*p*-Value	OR [95% CI]	*p*-Value	OR [95% CI]	*p*-Value	% Mediated ^1^
Polypharmacy	1.55 [1.35,1.79]	<0.001	1.47 [1.27,1.70]	<0.001	1.06 [1.02,1.10]	0.004	13.3
Cognitive impairment	1.53 [1.31,1.79]	<0.001	1.53 [1.31,1.79]	<0.001	1.00 [1.00,1.00]	0.842	NA
Sleep problems	1.55 [1.34,1.78]	<0.001	1.49 [1.29,1.71]	<0.001	1.04 [1.02,1.06]	<0.001	9.3
Pain	1.55 [1.34,1.78]	<0.001	1.40 [1.21,1.61]	<0.001	1.11 [1.07,1.14]	<0.001	23.5
Low handgrip strength	1.52 [1.30,1.78]	<0.001	1.51 [1.29,1.77]	<0.001	1.01 [1.00,1.01]	0.121	NA
Difficulty in ADL	1.54 [1.33,1.78]	<0.001	1.47 [1.27,1.70]	<0.001	1.05 [1.03,1.07]	<0.001	10.7
Obesity	1.51 [1.29,1.77]	<0.001	1.48 [1.27,1.74]	<0.001	1.02 [1.00,1.04]	0.014	5.1
Underweight	1.52 [1.30,1.77]	<0.001	1.52 [1.30,1.78]	<0.001	1.00 [1.00,1.00]	0.541	NA
All mediators	1.51 [1.29,1.77]	<0.001	1.26 [1.06,1.49]	0.007	1.20 [1.13,1.27]	<0.001	44.5

Abbreviations: OR, odds ratio; CI, confidence interval; ADL, activities of daily living. Models were adjusted for sex, age, education, marital status, alcohol consumption, and previous falls. Falls were those that were assessed at Wave 2 and referred to those that occurred since Wave 1. Mediating variables were assessed at baseline (i.e., Wave 1). ^1^ Percentage mediated was only calculated in the presence of a significant indirect effect (*p*-value < 0.050).

## Data Availability

Researchers interested in using TILDA data may access the data for free from the following sites: Irish Social Science Data Archive (ISSDA) at University College Dublin http://www.ucd.ie/issda/data/tilda/ (accessed on 5 August 2021); Interuniversity Consortium for Political and Social Research (ICPSR) at the University of Michigan.
